# Supplementary material for: Nanoparticle-Shielded dsRNA Delivery for Enhancing RNAi Efficiency in Cotton Spotted Bollworm Earias vittella (Lepidoptera: Nolidae)
Source: Int J Mol Sci. 2023 May 23;24(11):9161. doi: 10.3390/ijms24119161 (PMC10252998; doi:10.3390/ijms24119161)
Supplement: Supplementary file 1 [file ijms-24-09161-s001.zip › ijms-2307797-supplementary.pdf]

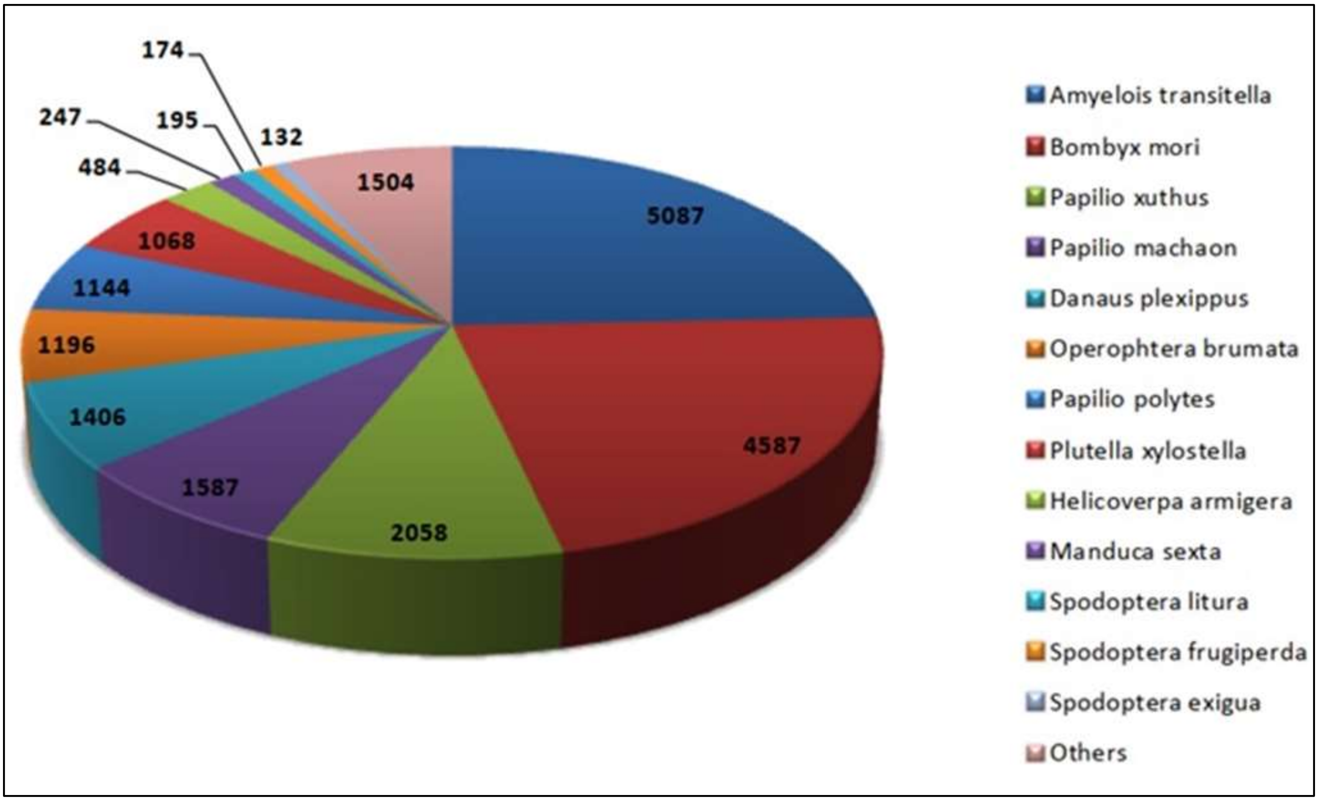

Figure S1. BLASTx homology search against the Nr database yielded the top hits for *E. vittella* sequences in insects and other taxa based on the highest score.

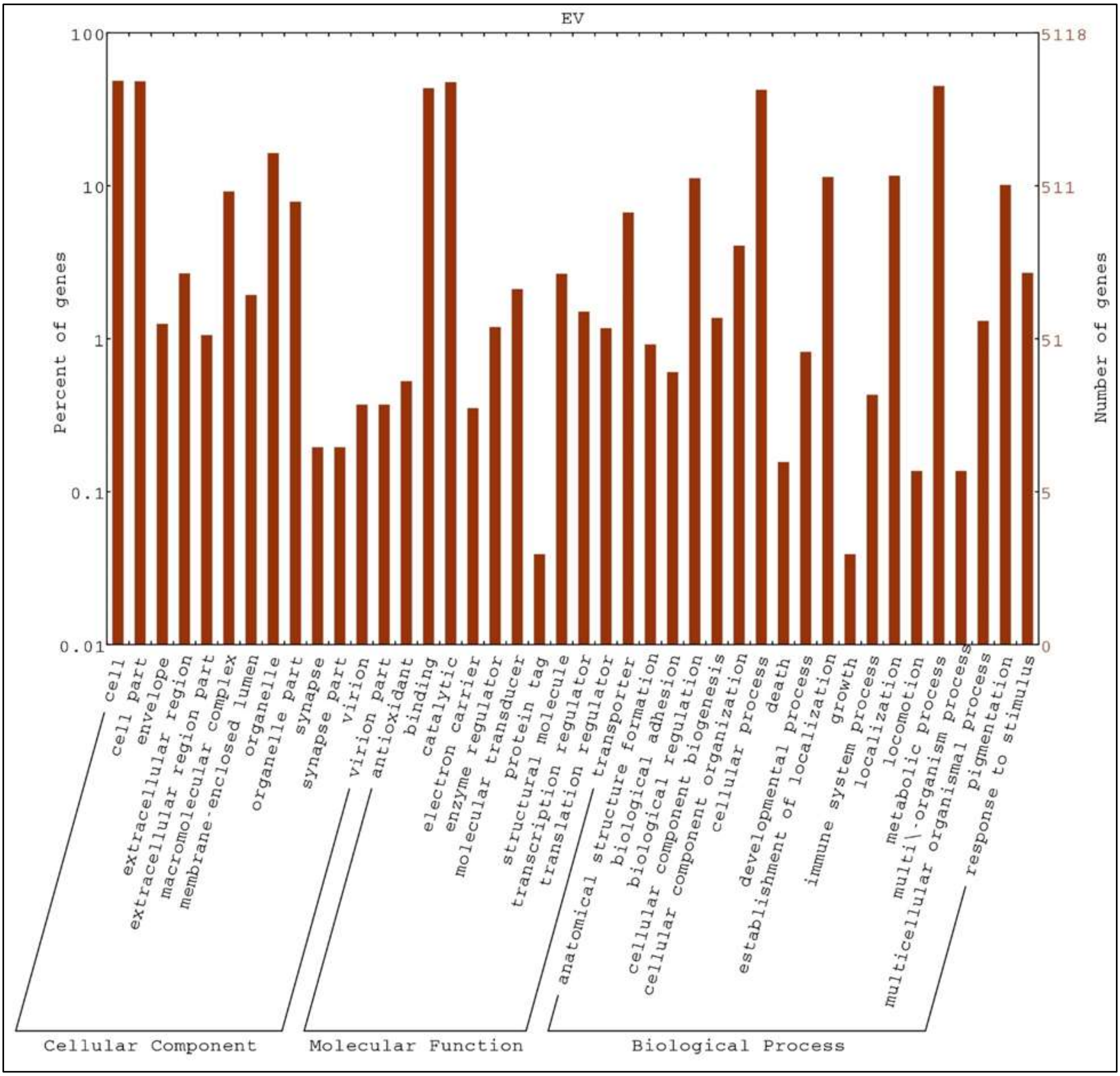

Figure S2. E. *vittella* transcripts classification based on anticipated gene ontology terms by WEGO plot connected to the cellular component, molecular function, and biological process.

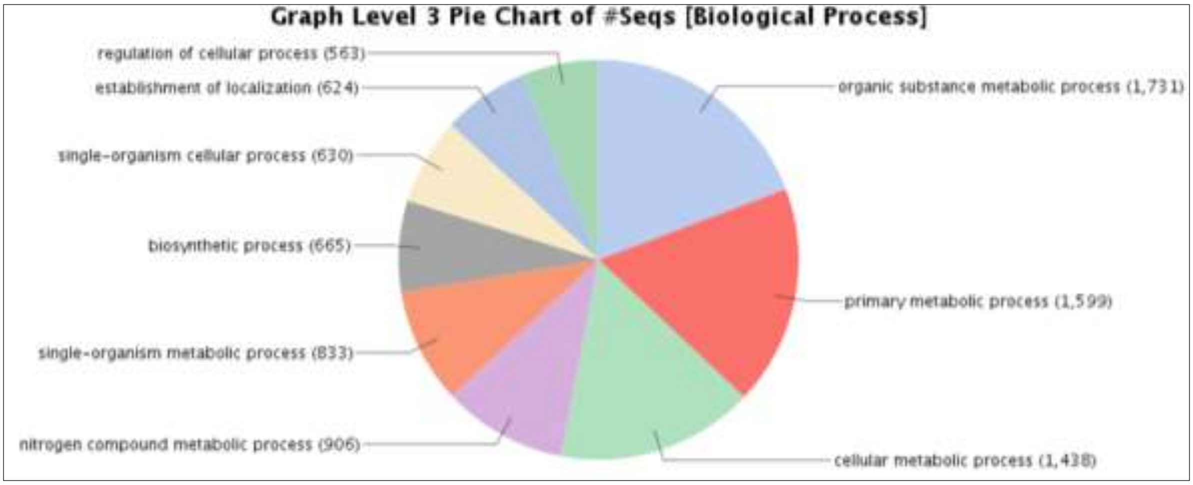

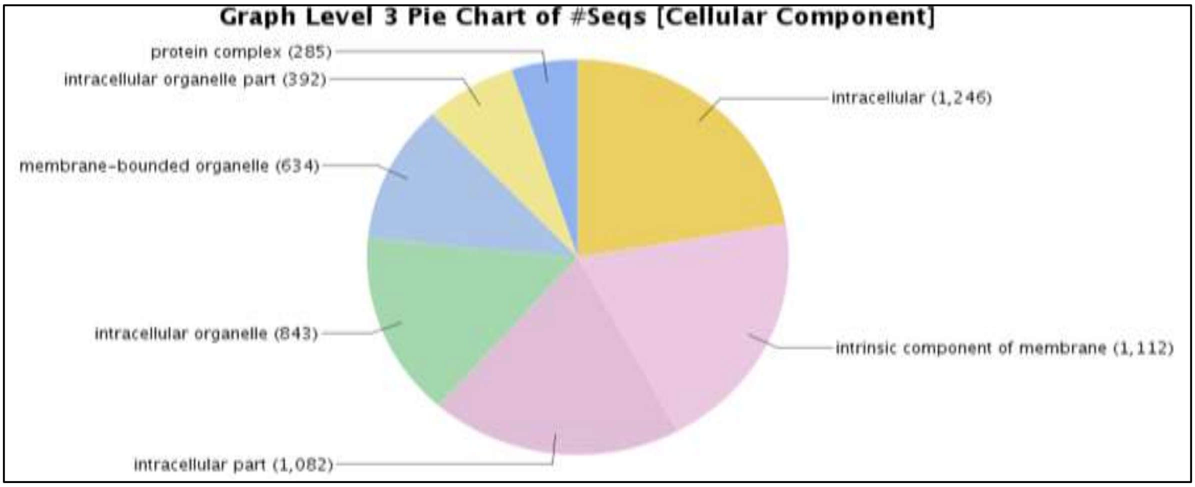

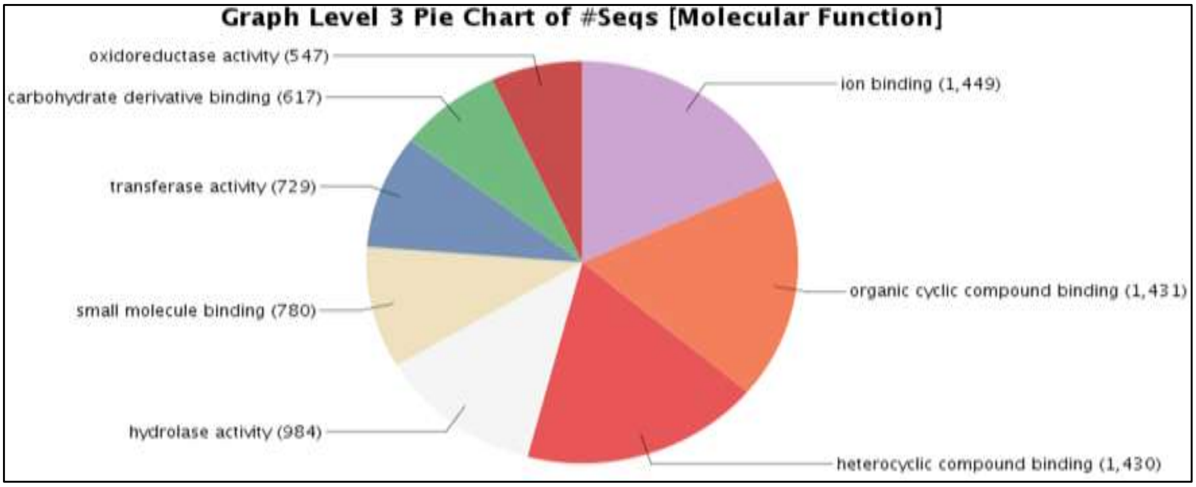

Figure S3. Categorization of *E. vitella* CDS according to KEGG pathways that connect to biological process, cellular components and molecular functions.

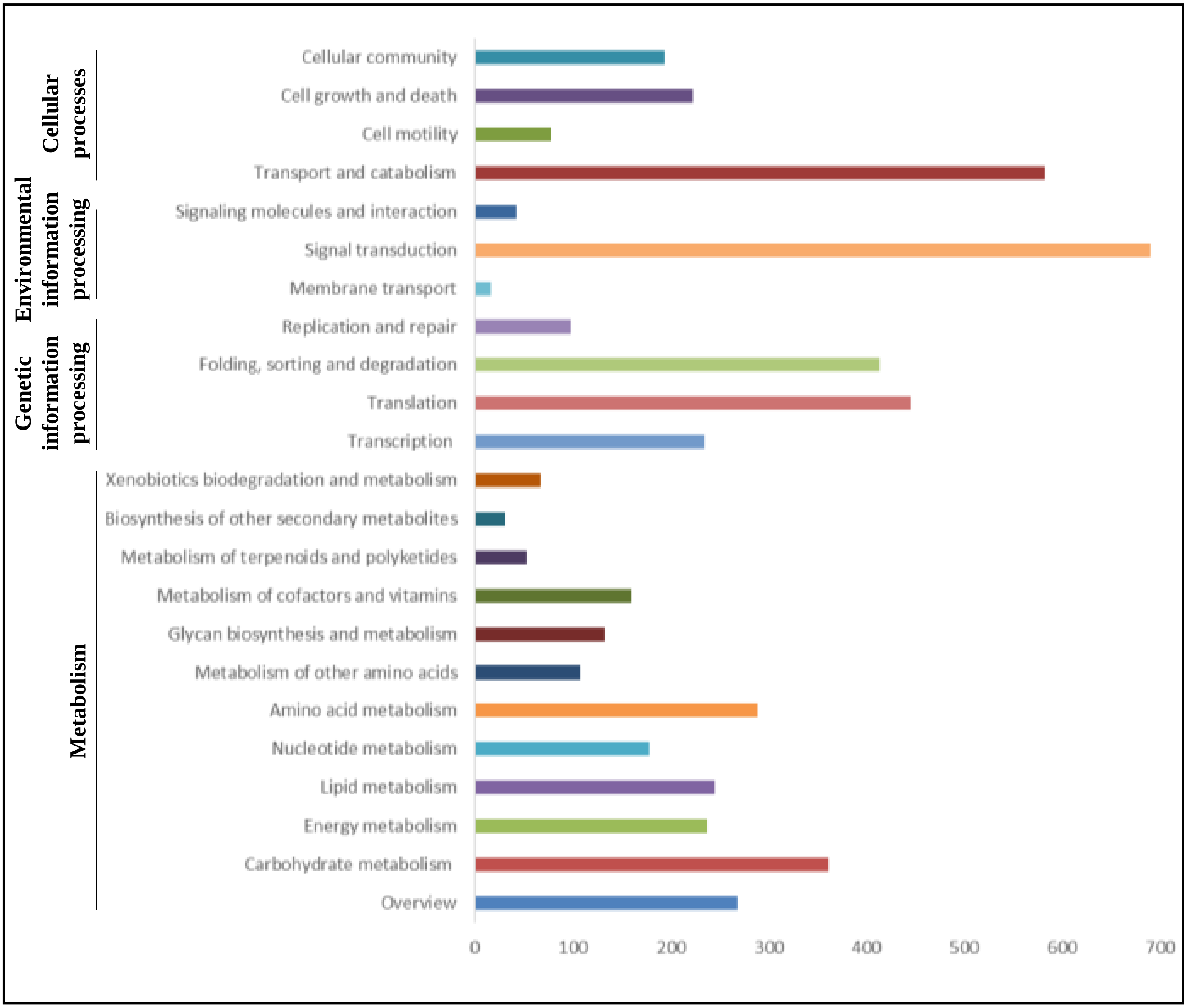

Figure S4. KEGG pathway classification of CDS of *E. vittella*.

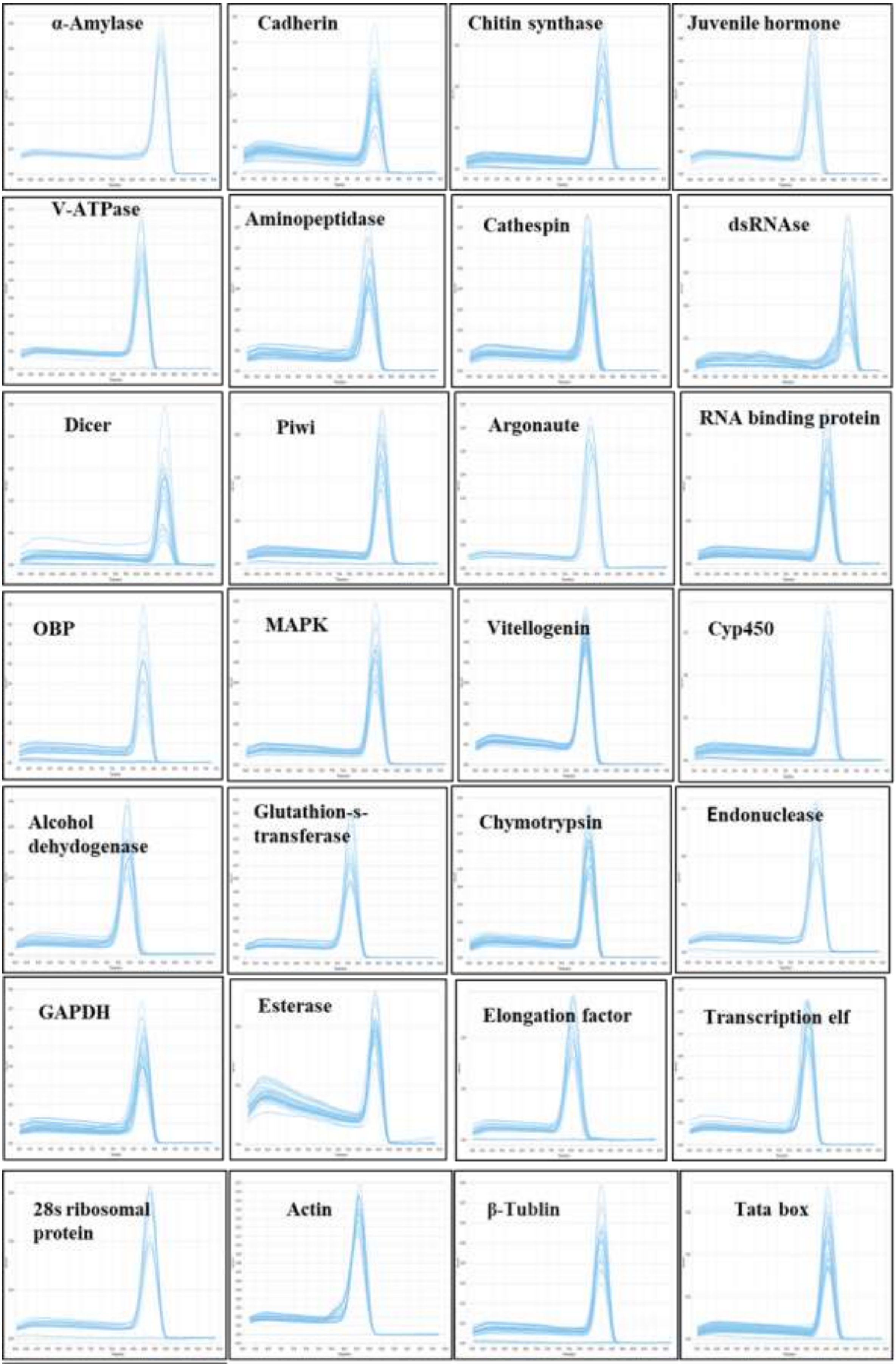

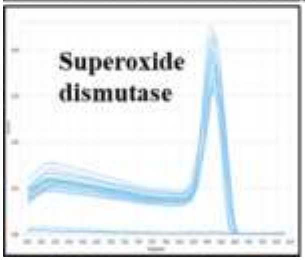

Figure S5. Melt curve analysis of different genes employed in the study to assess the specificity of primer to generate a single amplicon during RT-qPCR .

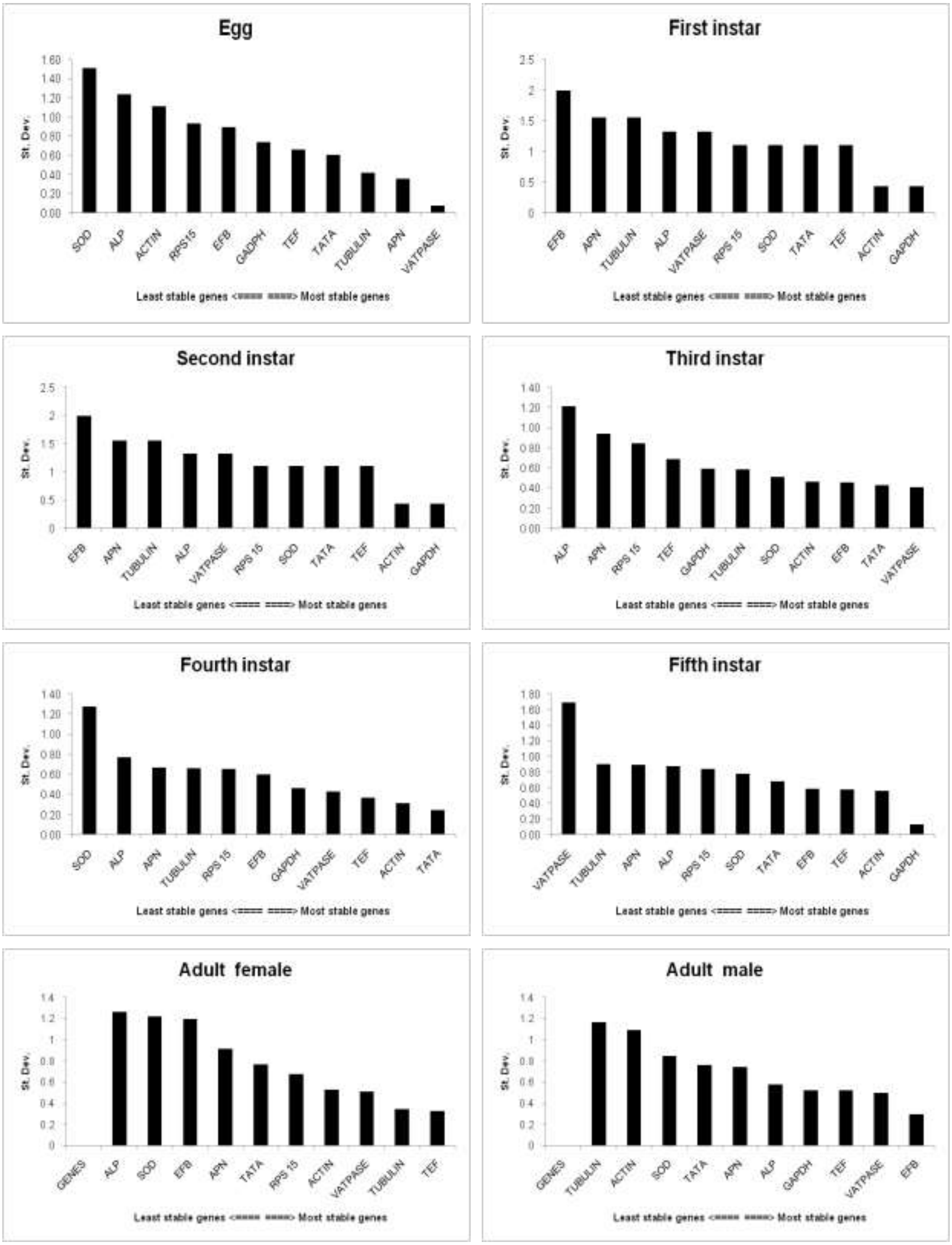

Figure S6. The standard deviation calculated using NormFinder for candidate reference genes across all developmental stages in *E. vittella*. The least stable genes were those with the greatest stability values, whereas the most stable genes were those with lower stability values.

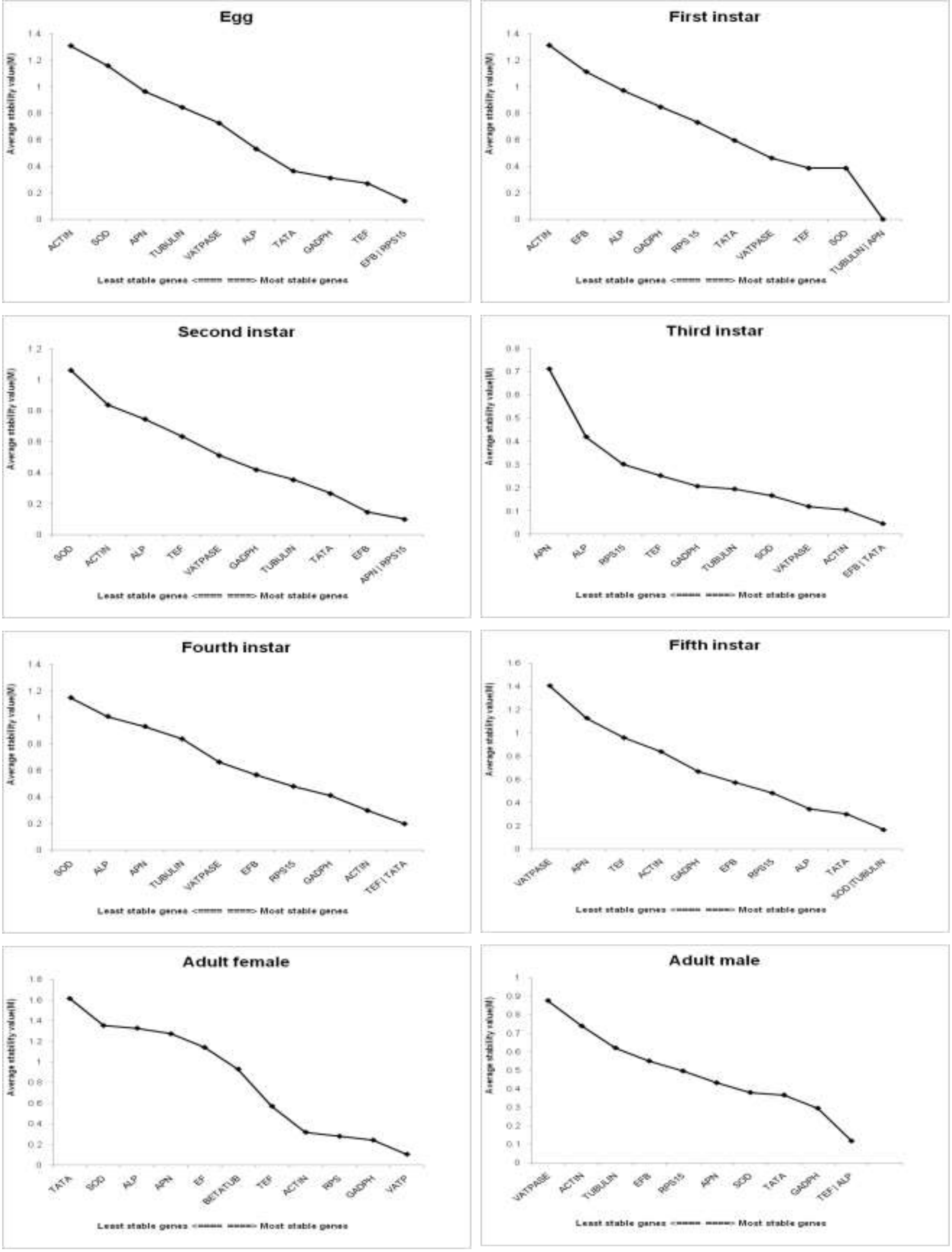

Figure S7. Stability and ranking of candidate reference genes of E. vittella calculated using expression stability values (M) in geNorm software. Gene expression stability graph based on stepwise exclusion method and average expression stability values (M-value). Gene stability is higher when the M-value is lower. The direction of the arrow indicates the most and least stable reference genes.

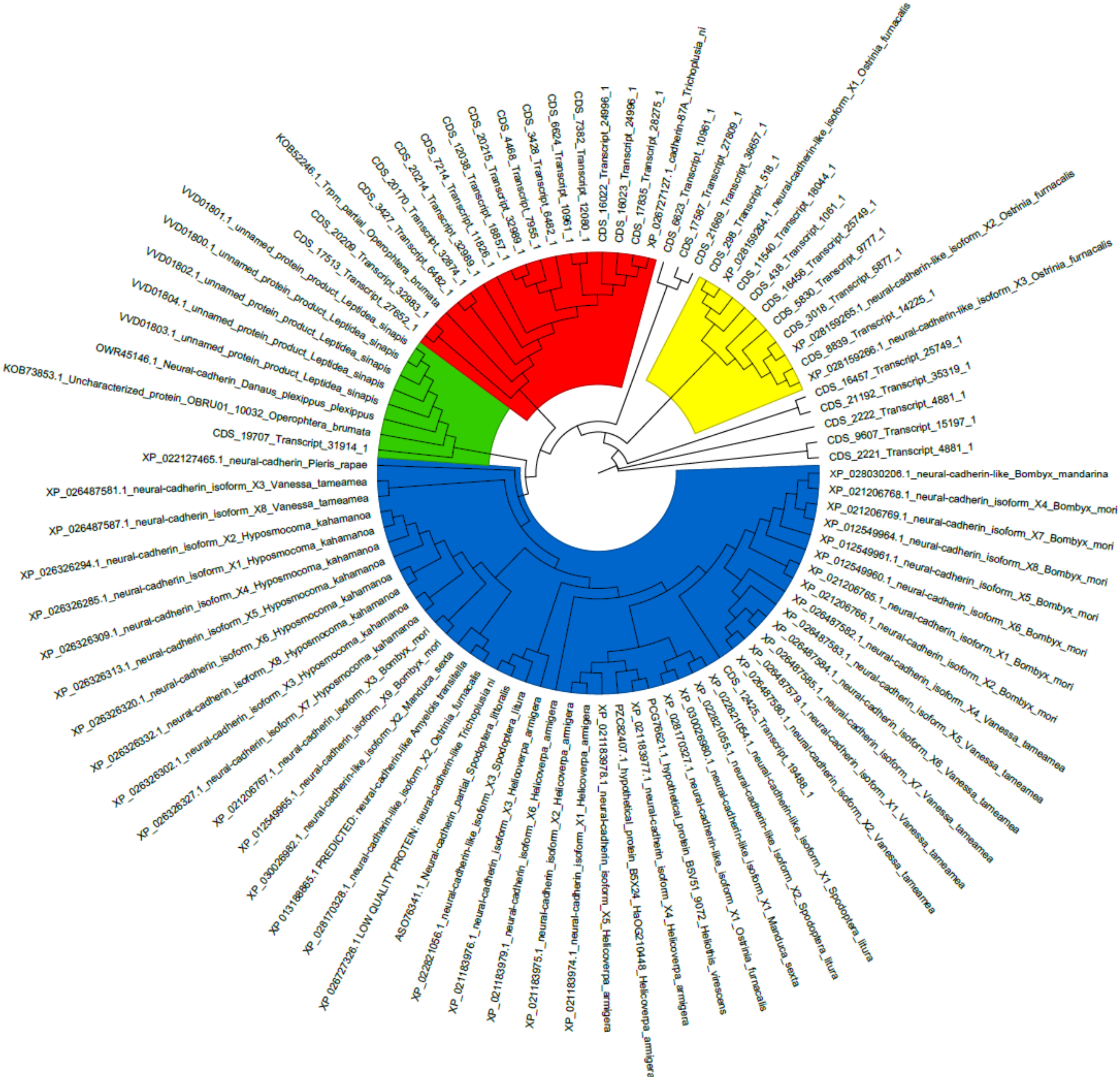

Figure S8 Phylogenetic analysis of cadherin like genes/ transcripts from *E. vittella* with other insect species using neighbour joining method of in MEGAX at 500 bootstrap value.

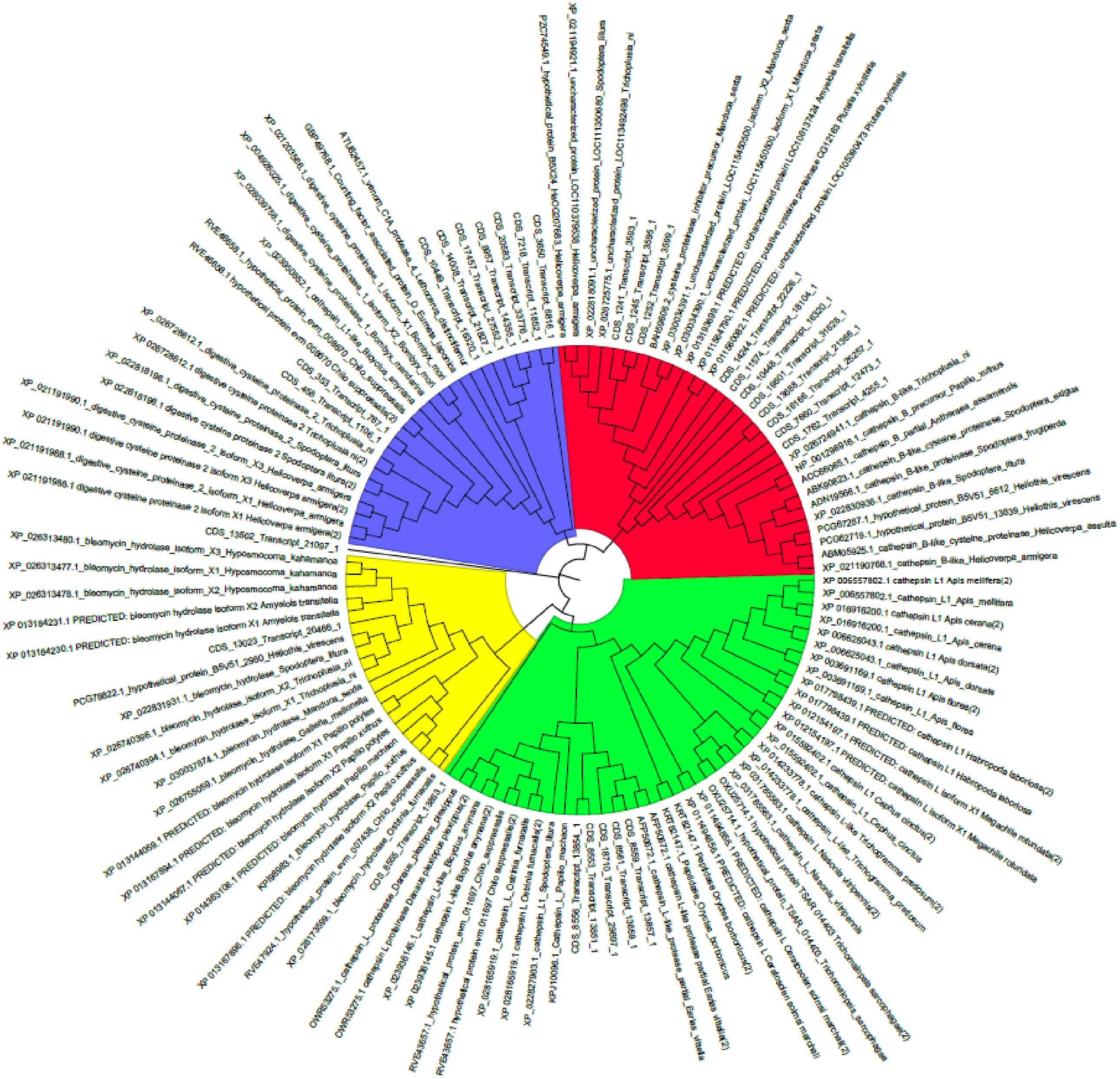

Figure S9 Phylogenetic analysis of cathepsin like genes/ transcripts from *E. vittella* with other insect species using neighbour joining method of in MEGAX at 500 bootstrap value.

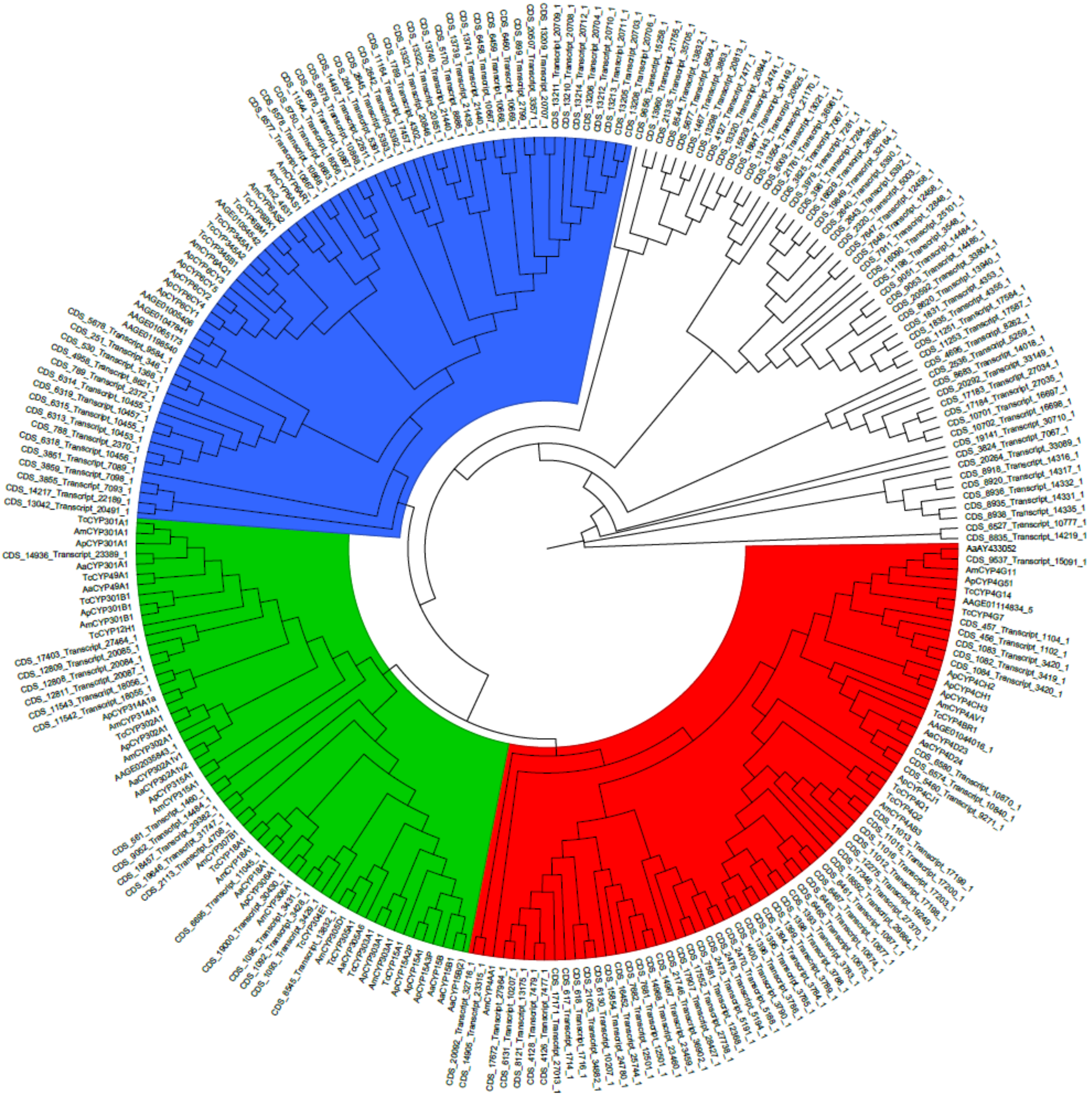

Figure S10 Phylogenetic analysis of E. vittella cytochrome P450s genes/ transcripts using neighbour joining method of in MEGAX at 500 bootstrap value with representative genes (with NCBI Acession numbers) from other insect species. Sequences in green color belong to CYP2 clan, blue coloured are from CYP3 clan, yellow coloured are from CYP4 clan and remaining could not be categorized.

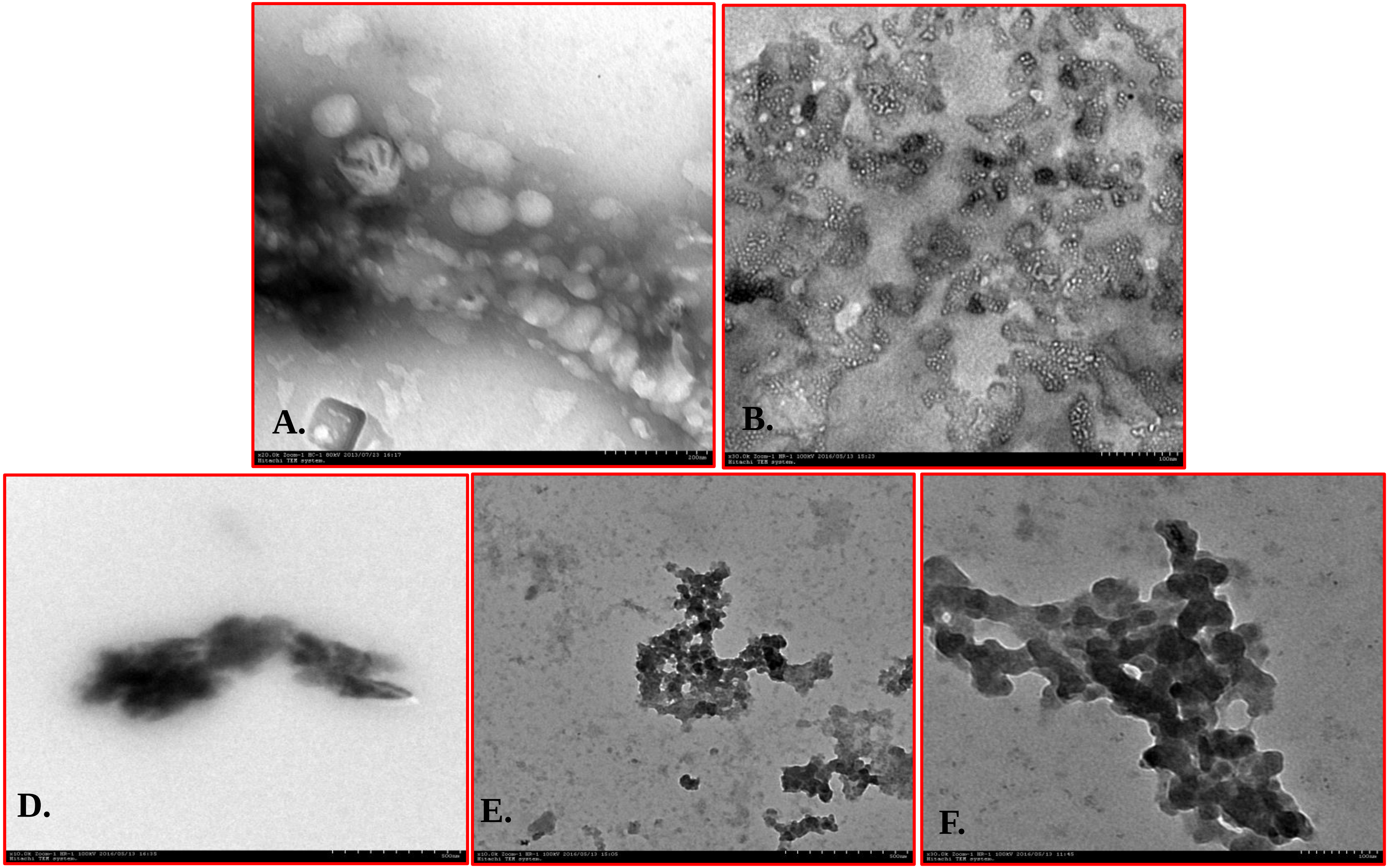

Supplementary Figure S11 Transmission Electron micrographs depicting the shape and dimensions (in nm) A. naked chitosan NPs (20.0 K magnification) B. dsRNA- Chitosan nanoconjugates (30.0K magnification) C. Naked CQD (10.0 K magnification) D. dsRNA-CQD nanoconjugates (10.0 K magnification), E dsRNA- CQD nanoconjugates (30.0 K magnification).

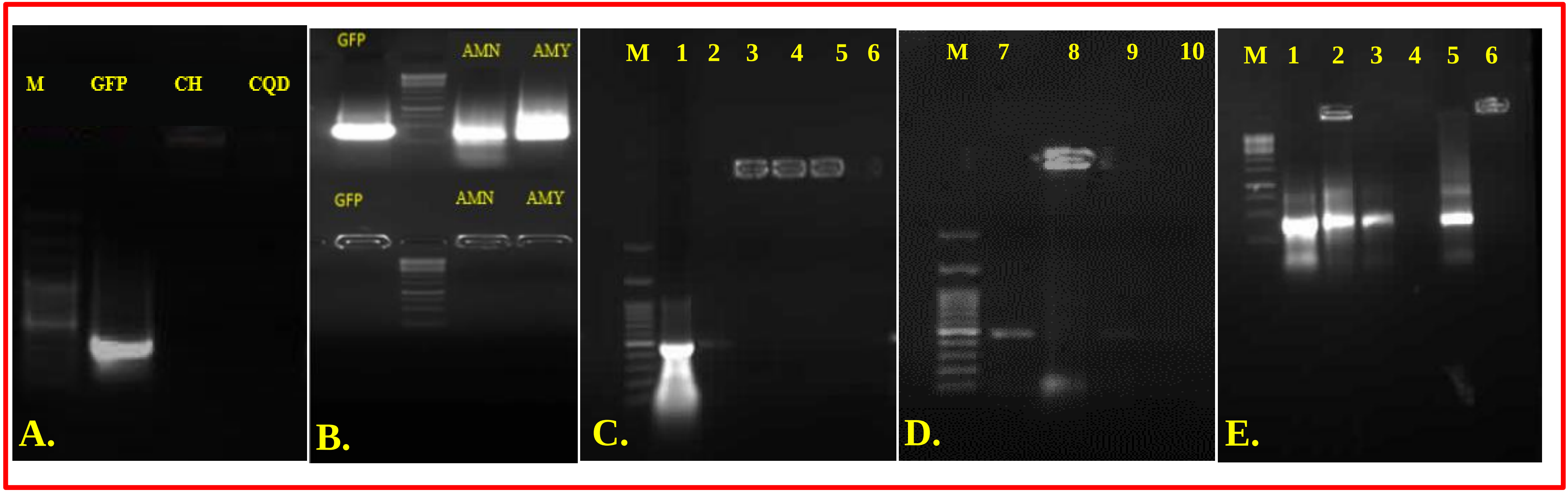

Supplementary Figure S12 Gel retardation assay for the confirmation of binding of dsRNA with Nanocarrirers A: Naked dsRNA of Green fluorescent protein (GFP); CH: dsGFP-chitosan nanoconjugate stuck in the well and CQD: dsGFP- CQD nanoconjugate stuck in the well B: Upper panel : Naked GFP dsRNA , Aminopeptidase (AMN) naked dsRNA and Alphaamylase (AMY) naked dsRNA Lower panel : GFP dsRNA with chitosan coating glowing in the well, AMNdsRNA with chitosan coating glowing in the well and AMYdsRNA with chitosan coating glowing in the well. C and D: Gel picture showing minimum concentration of chitosan required to bind efficiently to dsRNA 1. 1μg naked dsGFP; 2. 1μg dsGFP diluted in 100μl of double distilled water; 3. 1μg dsGFP in 100μl chitosan solution; 4. 1μg dsGFP in 50μl chitosan solution; 5. 1μg dsGFP in 25μl chitosan solution; 6. 1μg dsGFP in 12.5 μl chitosan solution; 7. 1μg dsGFP diluted in 100μl of double distilled water; 8. 1μg dsGFP in 6.25μl chitosan solution 9. 1μg dsGFP in 3.1 μl chitosan solution led to partial binding with dsRNA ; 10. 1μg GFP dsRNA in 1.6 μl chitosan solution unable to bind dsRNA . E: Gel picture showing minimum concentration of lipofectamine required to bind efficiently to 1. 10μg dsGFP in 2 μl water (Control); 2. 10μg dsGFP in 2 μl lipofectamine unable to bind dsRNA; 3. 1μg dsGFP in 2 μl water; 4. 1μg dsGFP in 2 μl lipofectamine stuck in the well showing conjugation of nanoparticle with dsRNA 5. 5μg dsGFP in 2 μl water (Control); 6. 5μg dsGFP in 2 μl lipofectamine stuck in the well showing conjugation of nanoparticle with dsRNA .

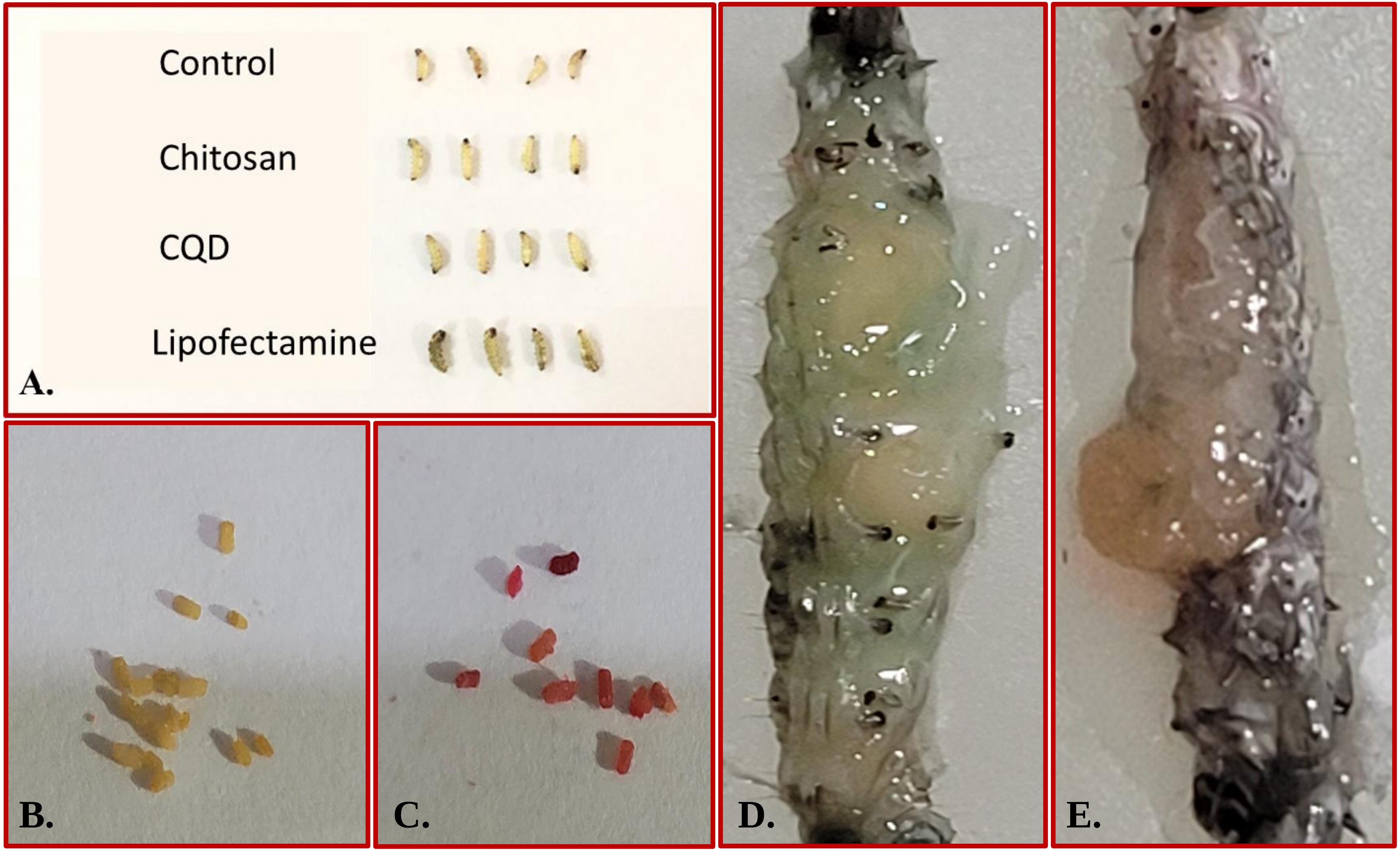

Supplementary Figure S13: A. Live *E. vittella* larvae after feeding of nanoparticles along with control B. Faecal matter of larvae fed with C. Faecal matter of larvae fed with red-dye labelled dsRNA-nanoparticles D . Midgut of larvae fed with control diet E. Midgut of larvae fed with red-dye labelled dsRNA-nanoparticles.

Table S1. *E. vittella* transcriptome analysis and statistical summary and BUSCO analysis.

**Statistical summary of RNA-seq**	
No. of reads	18,312,423
Read length	2 x 75
No. of bases	2,772,037,599
Total data in GB	2.7 GB
**Assembly statistics**	
No. of transcripts	37,025
Total transcripts (bases)	38,893,295
N50	1,576
Maximum transcript length	15,331
Minimum transcript length	200
Mean transcript length	1,050
**Summary of CDS Statistics**	
No. of CDS	21,782
Total CDS length (bases)	21,373,275
Maximum CDS length	9,927
Minimum CDS length	297
Mean CDS length	981
**Distribution of no. of transcripts according to their length**	
200 to 500 range of transcripts	13,038
500 to 1000 range of transcripts	9,869
1000 to 2000 range of transcripts	9,467
2000 to 3000 range of transcripts	3,045
3000 to 4000 range of transcripts	978
4000 to 5000 range of transcripts	388
≥5000 range of transcripts	240
**Distribution of no. of CDS according to their length**	
200 to 500 range of CDS	5,757
500 to 1000 range of CDS	8,200
1000 to 2000 range of CDS	6,216
2000 to 3000 range of CDS	1,112
3000 to 4000 range of CDS	339
4000 to 5000 range of CDS	85
≥5000 range of CDS	73
**Annotation distribution**	
No. of CDS	21,782
No. of CDS with Blast hit	20,869
No. of CDS without Blast hit	913
**BUSCO analysis of assembly completeness**	
BUSCO Notation	
Complete BUSCOs (C)	0.779

Complete and single-copy BUSCOs (S)	0.505
Complete and duplicated BUSCOs (D)	0.274
Fragmented BUSCOs (F)	0.14
Missing BUSCOs (M)	0.081
Total BUSCO groups searched (n)	1658

Table S2. List of reference genes evaluated in this study.

S. No.	Gene Name	Gene Symbol	Accession No.	Primer Sequence (5' → 3')	Amplicon Size (bp)	Tm (°C)	Amplicon Efficiency	Correlation Coefficient
1	*Actin-4*	*ACTIN*	AGR44798.1	F: GGACTTCGAGCAGGAGAT<br>R: GATTCCATACCCAAGAATGAGG	150	60	117.83	0.99
2	*Transcription elongation factor S-II*	*TEF*	XP_026756955.1	F: GCCGAAGAGTTAGAGGAATGTA<br>R: GTACGCAATGTAGGATTCTTAGG	119	60	123.9	0.97
3	*28S ribosomal protein S18b*	*RPS*	XP_022820459.1	F: GGACAAATATTGGACCCATTCA<br>R: CAAATGTGAGATAACCCTGGTC	103	60	102.58	0.99
4	*V-type proton ATPase subunit d*	*VATPASE*	XP_021184096.1	F: CTGTATCAGTGAGCAGGATTTG<br>R: TCACACATAACATCAGCAGTAG	132	60	115.21	0.81
5	*TATA box binding protein*	*TATA*	XP_026732972.1	F: GAGTTATTCCCTGGGCTTATTT<br>R: CTCTTACTTTGGCTCCTGTTAG	100	60	98.16	0.97
6	*Elongation factor 1-alpha*	*EFB*	AKP17624.1	F: GTATCTCCAAGAACGGACAGAC<br>R: CCTTCTTGATTTCCTCGAAACG	140	60	110.21	0.93
7	*Beta tubulin*	*TUBULIN*	NP_001036887.1	F: AATGAACACATACTCAGTCGTC<br>R: CCTCGTTGTCGATACAATAAGT	128	60	83.39	0.99
8	*Glyceraldehyde-3-phosphate dehydrogenase*	*GADPH*	AEB26314.1	F: GCATCTCTCTCAATGACAACTT<br>R: CTTGGTCTGGATGTACTTGATG	104	60	119.53	0.98
9	*Aminopeptidase N*	*APN*	AAP33526.1	F: CTACTCCAGATGTCAGCACTAC<br>R: CTAACGCTACAACAGACACAAG	129	60	91.3	0.78
10	*Alkaline phosphatase*	*ALP*	ALK86921.1	F: CAACATGCAAACTAACCCAGAG<br>R: CTCCCTCCACGAATAAGAAGAA	110	60	119.95	0.99
11	*Superoxide dismutase*	*SOD*	XP_028177871.1	F: AGACAGTCAGATTTCCCTCA<br>R: TGACACCGCATGCAATAC	146	60	122.14	0.98

Table S3. List of candidate genes primers evaluated in this study.

S. No.	Gene name	Gene Symbol	Primer Sequence (5'→3')	Amplicon Size (bp)
1	RNA binding protein	RBP	F: ATGGTGGTGGCGGTGATAA<br>R: ATAGCCCTGCTGAGCATACC	140
2	Dicer	Dicer	F: TGATCTTCGTTCGGCGTTAG<br>R: CCTTGTCGAGTTTCGGATTCA	107
3	Piwi	Piwi	F: CCGGGTGATTACCACTACATTC<br>R: TAATCACGGCCCATCAACTG	89
4	V-type proton ATPase subunit d	vATPase	F: CTGTATCAGTGAGCAGGATTTG<br>R: TCACACATAACATCAGCAGTAG	122
5	Aminopeptidase N	AMN	F: CTACTCCAGATGTCAGCACTAC<br>R: CTAACGCTACAACAGACACAAG	110
6	Cadherin	Cad	F: CACCTAATTACCCTGTC<br>R: CGGAATCTGTAGCTATG	109
7	Alpha amylase	Amy	F: ACGCTAGGCCTTACATCTACCA<br>R: GCTCATAATCTGAGGCTCTCCA	109
8	Cytochrome 450	Cyp450	F: TCCCTCATCCAAAACCGTTACC<br>R: CGTTGGGAAACTTCTTGCACAG	108
9	Glutathione S-transferase	GST	F: CGGTGGTAAAGATGGTGACGA<br>R: CGCTAGTTTGAAATCGCCCAC	129
10	Acyl-CoA dehydrogenase	ADH	F: GTTGGGTCACATCTGCCGTA<br>R: CCCTGACGCTGATGATAGGC	101
11	Multidrug resistance-associated protein lethal	MDRA	F: ACACAGACAGCGGAGAGAGT<br>R: CGCCACCAAGCCTATCAGAG	112
12	Chymotrypsin-1-like	Chymotrypsin	F: GTCGCCGGTGGTCAATACTC<br>R: GGCACATCAACAAGCACCAG	149
13	Esterase B1-like	Esterase	F: AGCTGACCAAAACCGACCAA<br>R: TCAGTCCACAGCTTCAACCG	103
14	Endochitinase A like	Endochitinase	F: AATTCATCCGTCCCAGCGTT<br>R: GGTGAGCTTGTTGGAGTCGA	136
15	Exonuclease	Exonuclease	F: CTAAGAAAAGGGCAGCTGAATTGT<br>R: GCGAGGGCCATTTTGTGAGT	100
16	Odorant binding protein	Odorant binding protein	F: TAAAGACCACCCCAGCCTCT<br>R: GCACAGTGGAAGCGAAACAG	111
17	Juvenile hormone methyl transferase	JHAMT	F: GCAGGATCCCGAGAAAGAAA<br>R: CTGGTATCTCGAACGGATTCAC	148
18	Vitellogenin	Vg	F: CGTTTCGCCTTATTCTACGGTTC<br>R: GTGTGCATGCCAGTGAAGTG	138

Table S4. List of dsRNA primers evaluated in this study.

S. No.	Gene Name	Gene Symbol	Primer Sequence (5'→3')	Amplicon Size
1	Cadherin	Cad	F: TAATACGACTCACTATAGCGTCACAGATGCTAATG<br>R: TAATACGACTCACTATAGGGATCGACTAGGTACAA	442
2	Aminopeptidase	AMN	F: TAATACGACTCACTATAGCTCTCTGACTCCATACT<br>R: TAATACGACTCACTATAGCATCCATCTCTTCTGAC	381
3	Alpha amylase	AMY	F: TAATACGACTCACTATAGACGCTAGGCCTTACATCTACCA<br>R: TAATACGACTCACTATAGGCTCATAATCTGAGGCTCTCCA	338
4	V-ATPase	V-ATPase	F: TAATACGACTCACTATAGTGATTGAGTTCCAGCATCTTAG<br>R: TAATACGACTCACTATAGTCACACATAACATCAGCAGTAG	394
5	Juvenile hormone methyl transferase	JHAMT	F: TAATACGACTCACTATAGGGGAACGGATATAAGCA<br>R: TAATACGACTCACTATAGGGTATCTCGAACGGATT	500
6	Chitin synthase	CHS	F: TAATACGACTCACTATAG<br>R: TAATACGACTCACTATAG	517

Table S5: Larval percentage survival post feeding with dsRNA nanoconjugates and nanoparticles post 72h feeding

**Percentage survival %**				
**Gene**	**Naked dsRNA**	**Chitosan coated dsRNA**	**CQD coated dsRNA**	**Lipofectamine coated dsRNA**
Aminopeptidase (AMN)	88.0 ± 4.89	84.0±4.0	84.0±4.0	80±6.3
Alpha amylase (AMY)	88.0± 4.89	88.0±4.89	84.0±4.0	84.0±4.0
Cadherin (CAD)	92.0±4.89	88.0±4.89	88.0±4.89	80.0±6.3
Juvenile hormone(JH)	84.0±4.0	84.0±4.0	96.0±4.0	84.0±4.0
Chitin synthase (CHS)	80.0± 6.3	64.0± 4.0*	76.0±4.0	72.0±4.89*
Vacuolar ATP (VATP)	96.0±4.0	92.0±4.89	88.0±4.89	84.0±4.0
**dsGFP (Control)**	96.0±4.0	92.0±4.9	88.0±4.9	88.0±4.9
**Control Set up**	**Semi synthetic (SS) diet**	**SS Diet+ Chitosan : PEI solution**	**SS Diet+ CQD Solution**	**SS Diet+ Lipofectamine**
	88.0 ± 4.89	92.0±4.89	92.0±4.89	88.0 ± 4.89

The data was analyzed using Student t-test, * indicates significant differences as compared to the respective dsGFP control ($p=0.05$). The data overrules any impact of nanoparticles on the survival of 4th instar larvae ($n=25$) of *E. vittella*.
